# Effect of Casein Phosphopeptide-amorphous Calcium Phosphate and Calcium Sodium Phosphosilicate on Artificial Carious Lesions: An *in vitro* Study

**DOI:** 10.5005/jp-journals-10005-1447

**Published:** 2017-02-27

**Authors:** Iqra Chaudhary, Abhay M Tripathi, Gunjan Yadav, Sonali Saha

**Affiliations:** 1 Postgraduate Student, Department of Pedodontics and Preventive Dentistry, Sardar Patel Post Graduate Institute of Dental & Medical Sciences Lucknow, Uttar Pradesh, India; 2 Professor and Head, Department of Pedodontics and Preventive Dentistry, Sardar Patel Post Graduate Institute of Dental & Medical Sciences Lucknow, Uttar Pradesh, India; 3 Reader, Department of Pedodontics and Preventive Dentistry, Sardar Patel Post Graduate Institute of Dental & Medical Sciences Lucknow, Uttar Pradesh, India; 4 Reader, Department of Pedodontics and Preventive Dentistry, Sardar Patel Post Graduate Institute of Dental & Medical Sciences Lucknow, Uttar Pradesh, India

**Keywords:** Calcium sodium phosphosilicate, Casein phos-phopeptide-amorphous calcium phosphate, Demineralization, Fluoride, Remineralization, Scanning electron microscopy and energy-dispersive X-ray analysis

## Abstract

**Aim:**

To compare new remineralizing agents calcium sodium phosphosilicate paste and casein phosphopeptide-amorphous calcium phosphate (CPP-ACP) paste with that of fluoridated toothpaste in remineralization of early carious lesions using scanning electron microscopy and energy-dispersive X-ray (SEM-EDX) analysis.

**Materials and methods:**

Sixty sound extracted premolars were collected and placed in demineralizing solution for 4 days to produce artificial carious lesions. All specimens were evaluated for any loss of mineral content using SEM-EDX analysis. Samples were randomly assigned to three groups: Group I: Fluoridated toothpaste (control), group II: CPP-ACP paste, and group III: Calcium sodium phosphosilicate paste. Specimens were then treated with above-mentioned remineralizing agents and again measured for mineral content using SEM-EDX analysis.

**Results:**

Group III (calcium sodium phosphosilicate paste) showed highest significant difference followed in descending order by group II (CPP-ACP paste) and group I (fluoridated toothpaste).

**Conclusion:**

Calcium sodium phosphosilicate paste showed maximum remineralizing potential compared with CPP-ACP and fluoridated toothpastes.

**How to cite this article:**

Chaudhary I, Tripathi AM, Yadav G, Saha S. Effect of Casein Phosphopeptide-amorphous Calcium Phosphate and Calcium Sodium Phosphosilicate on Artificial Carious Lesions: An *in vitro* Study. Int J Clin Pediatr Dent 2017;10(3):261-266.

## INTRODUCTION

Dental caries is a pathological process of localized destruction of tooth tissue by microorganisms. There are many possibilities to intervene in this continuing process to arrest or reverse the progress of the lesion via remineraliza-tion. The noninvasive treatment of early caries lesions by remineralization has the potential to be a major advancement in the clinical management of the disease. Therefore, the best strategy for caries management is to focus on the methods of improving the remineralizing process with the aid of various remineralization products.^1^

Over the last few decades, fluoride is known to promote remineralization, but is dependent on calcium and phosphate ions from saliva to accomplish this process. Recent investigations have primarily focused on various calcium phosphate-based technologies which are designed to supplement and enhance fluoride’s ability to restore tooth mineral.

Recently, bioactive glass materials have been introduced in many fields of dentistry. Bioactive glass is considered to be a breakthrough in remineralization technology. It is a multicomponent inorganic compound made up of elements, such as silicon, calcium, sodium, and phosphorus. This compound in an aqueous environment releases bioactive calcium, sodium, and phosphate ions contributing to the remineralization process.^2^

Another calcium phosphate remineralization technology based on CPP-ACP has also been recently developed, where CPP stabilizes high concentrations of calcium and phosphate ions, together with fluoride ions, at the tooth surface by binding to pellicle and plaque, thus preventing demineralization and enhancing remineralization.^3^

Thus the aim of the present study was to compare the efficacy of new remineralizing agents—calcium sodium phosphosilicate paste and CPP-ACP—with that of fluoride-containing toothpaste in remineralization of artificial carious lesions using scanning electron microscope with SEM-EDX analysis.

## MATERIALS AND METHODS

In the present study, 60 sound premolar teeth extracted for the purpose of orthodontic treatment were collected from the Department of Oral and Maxillofacial Surgery, Sardar Patel Post Graduate Institute of Dental & Medical Sciences, Lucknow, Uttar Pradesh, India, and various private dental clinics.

### Lesion Formation

**Fig. 1:**
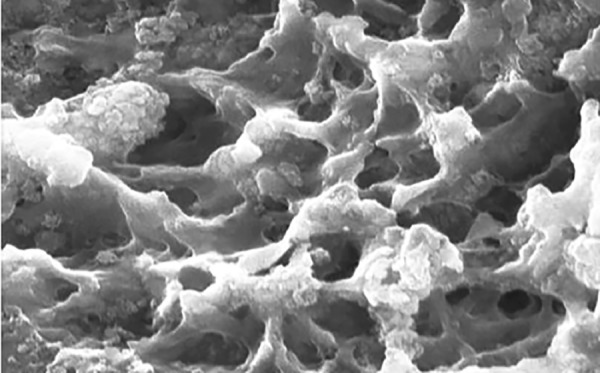
Structural analysis of demineralized enamel sample by SEM

**Graph 1:**
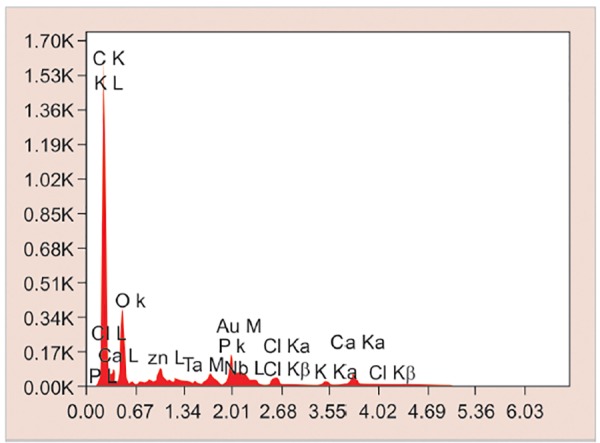
Elemental analysis of demineralized enamel sample by SEM-EDX analysis

Teeth were cleansed of soft tissue debris and inspected for cracks, hypoplasia, and white spot lesions. The teeth were then coated with a nail varnish, leaving a narrow window (4 mm × 1 mm wide), on the sound, intact surface of the buccal enamel. Each tooth was subsequently immersed in the demineralizing solution for 4 days to produce lesions. The buffered demineralizing and remineralizing solutions were prepared. All specimens were evaluated for any loss of mineral content (wt.%) using SEM-EDX on the fifth day (Fig. 1 and Graph 1).

### Preparation of Demineralizing Solution

The demineralizing solution, which contained 2.2 mM CaCl_2_, 2.2 mM KH_2_PO_4_, 0.05M acetic acid had a pH adjusted to 4.4 with 1 M KOH.

**Fig. 2:**
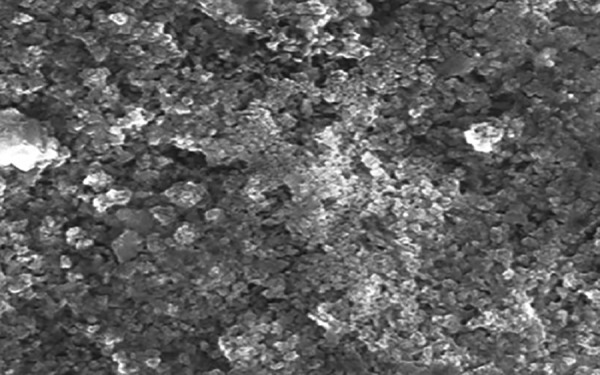
Structural analysis of remineralized enamel sample treated with fluoridated toothpaste by SEM

**Graph 2:**
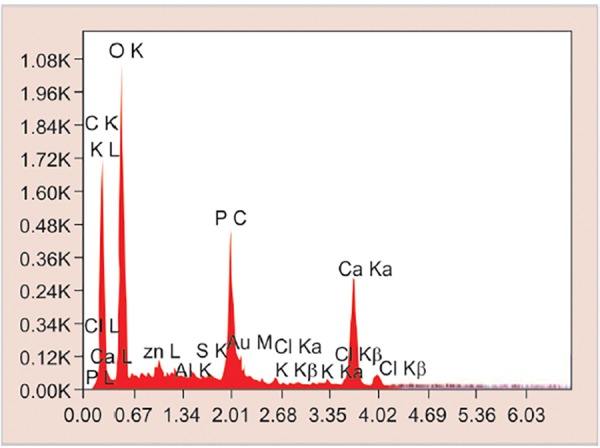
Elemental analysis of remineralized enamel sample treated with fluoridated toothpaste by SEM-EDX analysis

### Test Groups

Sixty specimens were randomly assigned to three treatment groups (20 in each group) as follows:

- *Group I:* Fluoridated toothpaste (positive control group)
- *Group II:* CPP-ACP toothpaste
- *Group III:* Calcium sodium phosphosilicate paste.

Specimens of each group were treated with the above-mentioned remineralizing agents for 7 days twice daily for 3 minutes followed by incubation in artificial saliva at 37°C.

### Preparation of Remineralizing Solution

The remineralizing solution, which contained 1.5 mM CaCl_2_, 0.9 mM NaH_2_PO_4_, 0.15 M KCl, had a pH of 7.0. This solution approximated to the super saturation of apatitic minerals found in saliva.

**Fig. 3:**
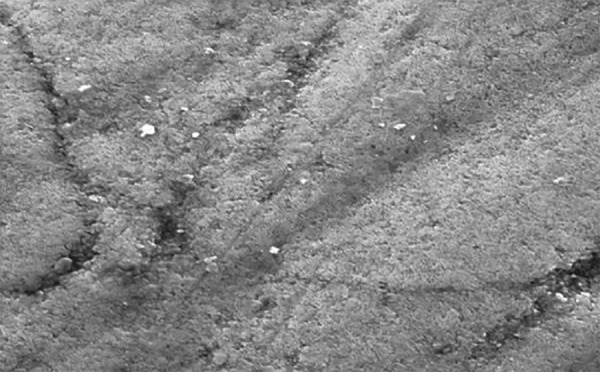
Structural analysis of remineralized enamel sample treated with CPP-ACP paste by SEM

**Fig. 4:**
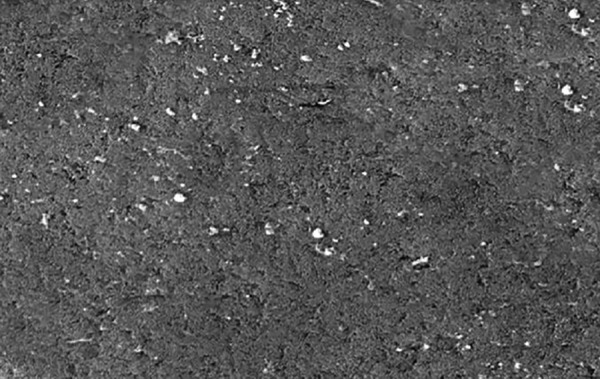
Structural analysis of remineralized enamel sample treated with calcium sodium phosphosilicate paste by SEM

**Graph 3:**
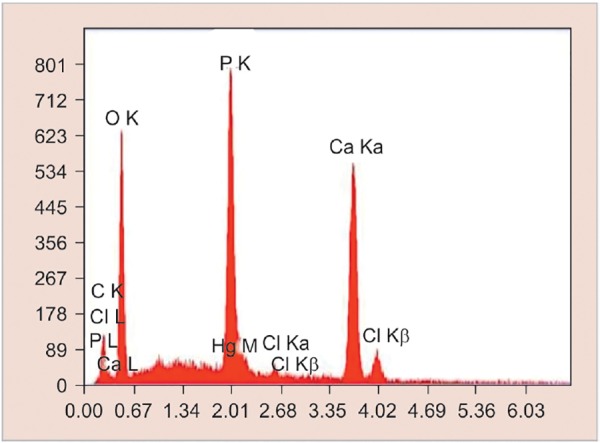
Elemental analysis of remineralized enamel sample treated with CPP-ACP paste by SEM-EDX analysis

**Graph 4:**
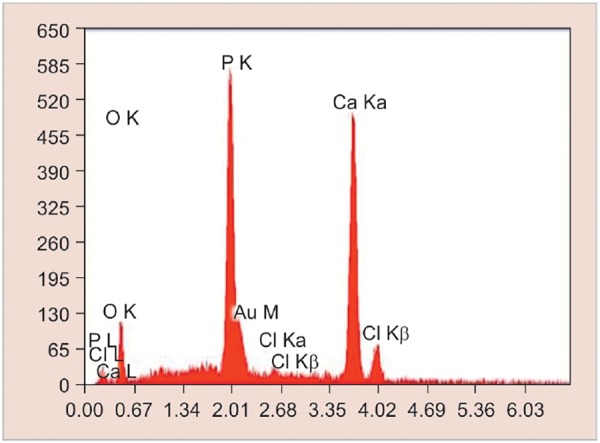
Elemental analysis of remineralized enamel sample treated with calcium sodium phosphosilicate paste by SEM-EDX analysis

The SEM-EDX analysis was done to measure the mineral content after the remineralization process (Figs 2 to 4 and Graphs 2 to 4).

### Evaluation Techniques

All the specimens were collected and analyzed under SEM-EDX analysis. It was used to determine calcium and phosphorus content in percentage weight of sound demineralized and remineralized enamel in each group.

### Statistical Analysis

Data were analyzed using Statistical Package for the Social Sciences. As the sample size was small, hence, normality assessment was done using Kolmogorov-Smirnov test. As a number of distributions lacked normality, a nonparametric evaluation plan was adopted. Data had been depicted as mean, median, and standard deviation.

## RESULTS

**Graph 5:**
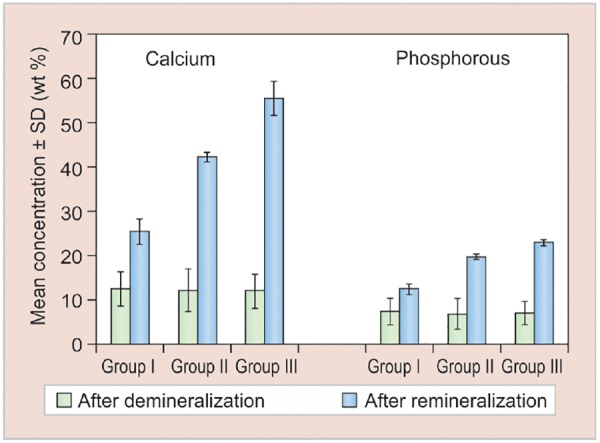
Change in calcium and phosphorous concentrations between demineralization and remineralization cycles in the three study groups. SD: Standard deviation

**Graph 6:**
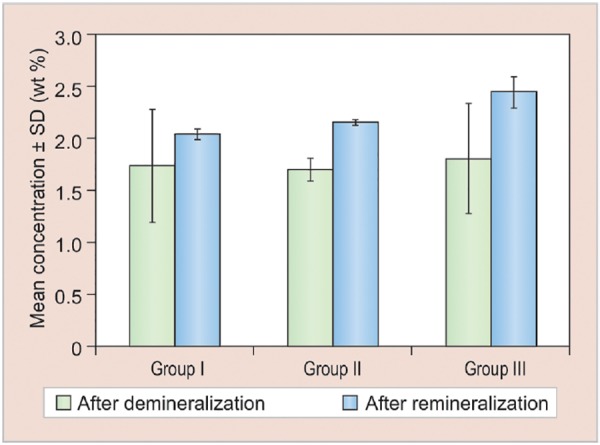
Change in Ca/P ratios between demineralization and remineralization cycles in the three study groups. SD: Standard deviation

Table 1, Graphs 5 and 6 show comparison of change in calcium and phosphorous concentrations and Ca/P ratios between demineralization and remineralization cycles in the three study groups, which revealed that an increase in calcium and phosphorous concentrations and Ca/P ratios was observed in all the three groups.

Based on the above evaluation, the concentration of calcium, phosphorus, and Ca/P ratios after remineral-ization observed in different study groups were in the following order:

Group III > Group II > Group I

## DISCUSSION

Dental caries is a transmissible bacterial disease caused by acids mainly lactic acid, formic acid, and propionic acid from bacterial metabolism (mutans *streptococci* and *lactobacilli* species) diffusing into enamel and dentin and dissolving the mineral. If this process progresses long enough at or below pH 5.5, the end result is a cavity.

When the pH rises, the reverse takes place, resulting in remineralization by deposition of calcium, phosphate, and fluoride ions in the form of fluorapatite, which are more resistant to dissolution by organic acids. Therefore, the best strategy for caries management is to focus on the methods of improving the remineralizing process with the aid of remineralization products.^4^

**Table 1:** **Table 1:** Comparison of change in calcium and phosphorous concentrations and Ca/P ratios between demineralization and remineralization cycles in the three study groups

				*After demineralization*				*After remineralization*				*Change*				*Significance of change (Wilcoxon signed rank test)*			
*Groups*		*n*		*Mean*		*SD*		*Mean*		*SD*		*Mean*		*SD*		*z*		*p*	
*Calcium*																			
I		20		12.26		3.79		25.14		2.70		12.89		3.64		3.920		<0.001	
II		20		11.91		4.81		41.97		1.11		30.06		5.41		3.920		<0.001	
III		20		11.82		3.81		55.27		3.75		43.45		4.94		3.920		<0.001	
*Phosphorous*																			
I		20		7.14		2.99		12.27		1.24		5.14		2.80		3.920		<0.001	
II		20		6.61		3.51		19.50		0.64		12.89		3.74		3.920		<0.001	
III		20		6.72		2.67		22.69		0.67		15.97		2.90		3.920		<0.001	
*Ca/P Ratio*																			
I		18		1.73		0.54		2.04		0.05		0.31		0.54		2.940		0.003	
II		17		1.70		0.11		2.15		0.03		0.45		0.12		3.621		<0.001	
III		19		1.80		0.53		2.44		0.15		0.64		0.52		3.058		<0.001	

SD: Standard deviation

Fluoride is the most commonly used remineralizing agent. When the acid attacks the enamel surface, the pH begins to rise, new and larger crystals that contain more fluoride (fluorhydroxyapatite) form, thereby reducing the enamel demineralization by forming fluorhydroxyapa-tite crystals and enhancing remineralization. Normally, remineralization by fluoride is a self-limiting surface phenomenon that prevents penetration of ions into the depth of the lesion. Additionally, fluoride might be highly effective on smooth surface caries but its effect is limited on pit and fissure caries. Overexposure of fluoride can also cause fluorosis. Hence, all these limitations have prompted researchers to search for nonfluoridated alternatives that aid in remineralization.^5^

A variety of calcium phosphate-based remineralization systems have now been commercialized to overcome the limited bioavailability of calcium and phosphate ions for the remineralization process.^1^ So far, none of the remineralizing agents have been found to be completely efficient; therefore, attempts have been made to find effective anticariogenic and remineralizing agents to be used in pediatric patients.

As several studies have demonstrated that milk-based products appeared to have anticaries properties in animal models, attention was focused on identifying the specific milk-based agents that were responsible for anticaries effect. The CPP-ACP nanocomplexes are derived from bovine milk protein, casein, and calcium and phosphate. They act by localizing ACP on the tooth surface, which buffers the free calcium and phosphate ion activities, thereby helping to maintain a state of supersaturation with respect to tooth enamel and thus preventing demin-eralization and enhancing remineralization.^3^

Another notable remineralizing agent containing calcium and phosphorus is a particulate bioactive glass, in the chemical form of calcium sodium phosphosilicate. When this particle comes in contact with saliva and water, it reacts and releases calcium and phosphate ions and binds to the tooth surface to form a layer of calcium phosphate. This layer then crystallizes into hydroxycarbonate apatite (HCA), resulting in the remineralization of teeth.^6^

Modern prospective caries study requires the measurement of small changes in a tooth’s mineral content, especially in a single caries lesion. Several methods have been proposed to evaluate demineralization, remineral-ization, surface defects, and degradation of sound enamel. Among them, the most commonly used method is SEM, which is most widely used to determine the morphological variation between the treated and untreated surface. But the major disadvantage of using SEM alone is that it does not evaluate the chemical composition as well as the amount of mineral loss and gain of the treated and untreated surfaces. Hence, the EDX component of the system is used in conjunction with SEM analysis, wherein SEM does the structural analysis and the elemental analysis is done by EDX.^4^

Thus the aim of the present study was to compare the efficacy of new remineralizing agents calcium sodium phosphosilicate paste and CPP-ACP with that of fluoride containing toothpaste in remineralization of artificial carious lesions using SEM-EDX analysis.

In the present study, human extracted teeth were preferred as they simulate the oral environment.^7^ The teeth were then coated with a nail varnish, leaving a narrow window, approximately 4 mm xl mm wide, on the sound, intact surface of the buccal enamel. According to Shirahatti et al^8^ and Vashisht et al,^7^ window of specific dimension was created to standardize the size of the window in all the specimens and to limit the area of paste application and to produce lesions only in window area. This would also help in lesion depth measurement compared with unaffected area covered by nail varnish. Each tooth was subsequently immersed in demineralizing solution for 4 days to produce lesions. All specimens were evaluated for any loss of mineral content (wt.%) using SEM-EDX on the fifth day.

Specimens of each group were treated with the mentioned remineralizing agents for 7 days twice daily for 3 minutes, followed by incubation in remineralizing solution at 37°C. The SEM-EDX analysis was done to measure the mineral content after the remineralization process.

On comparing the calcium and phosphorous concentrations and their ratios in the three study groups after remineralization, results of the present study revealed that mean values of group III (calcium sodium phospho-silicate paste) were maximum, followed in a descending order by group II (CPP-ACP paste) and group I (fluoridated toothpaste). Our results were also in accordance with the study performed by Alaudin and Fontana.^9^ This could be attributed to the continuous release of calcium and phosphate ions into the local environment for many days, which act as reservoirs for these ions.^10^ In *in vitro* studies performed by Narayana et al, calcium sodium phosphosilicate paste has been shown to release ions and transform into HCA for up to 2 weeks. This mechanism is found to be lacking in CPP-ACP and fluoride pastes.^2^ Another factor proving better remineralizing potential of these recent remineralizing agents could be the ionic reactions and resultant surface modifications beneficially interacting with collagen, allowing for strong bonding between collagen and the surfaces of these materials. This allows calcium sodium phosphosilicate particles to attach and remain on the dentin surface for a longer period of time and act as a reservoir for the continued long-term release of calcium and phosphate ions into the local environment as stated by Orefice et al.^11^

The results of the present study also revealed that CPP-ACP showed better remineralization potential than fluoride. Our results were also in accordance to the study performed by Oshiro et al.^12^ This may be due to the role of CPPs, which act as ACP carrier by localizing the highly soluble calcium phosphate phase at the tooth surface. A study conducted by Fahad and Al-Weheb^13^ also stated that CPP-ACP showed greater amount of remineraliza-tion in enamel than that of fluoride because of more bio-available calcium and phosphate ions in CPP-ACP agents. According to Yimcharoen et al, CPP-containing toothpaste had the same efficacy for inhibiting demineral-ization progression in primary teeth enamel as a 260 ppm fluoride-containing toothpaste minimizing the potential risk of fluorosis.^14^ Thus, CPP-containing toothpaste may be used for preventive treatment of caries in children who are allergic or sensitive to fluoride.

The results of the present study also revealed that group I (fluoridated toothpaste) showed the least rem-ineralization potential compared with group III (calcium sodium phosphosilicate paste) and group II (CPP-ACP paste). The results of the present study were in accordance to the studies conducted by Reynolds^1^ and Lata et al,^5^ who stated that comparatively lower remineralization potential of fluoride than the other study groups could be due to fluoride’s self-limiting surface phenomenon that prevented further penetration of calcium and phosphate ions into the depth of the lesion after the formation of fluorapatite. This is attributed to the fact that for every 2 fluoride ions, 10 calcium ions and 6 phosphate ions are required to form one unit cell of fluorapatite. Hence, the availability of calcium and phosphate ions can be the limiting factor for net remineralization to occur as stated by Reynolds et al.^1^ A study conducted by Yimcharoen et al also stated that fluoride might be highly effective on smooth surface caries but its effect is limited on pit and fissure caries. Hence, it does not confer absolute protection to dental caries. Another factor that limits the use of fluoride is its overexposure which causes fluorosis, as stated by Yimcharoen et al. Due to the potential risk of fluorosis, fluoride-containing toothpaste should be used with caution in small children who are unable to expectorate toothpastes or who are allergic to fluoride as stated by Yimcharoen et al.^14^

Hence, with the advent of remineralization therapies (calcium sodium phosphosilicate and CPP-ACP) and their various benefits over fluoride, these agents can be used as an adjunct to fluoride or independent to it.

## CONCLUSION

On comparing the calcium and phosphorous concentrations and their ratios in the three study groups after remineralization, calcium sodium phosphosilicate paste showed maximum remineralization potential followed in a descending order by CPP-ACP paste and fluoridated toothpaste respectively, apart from its benefits to reduce overexposure of fluoride and its side effects.
